# 22q11.2 duplication: a review of neuropsychiatric correlates and a newly observed case of prototypic sociopathy

**DOI:** 10.1101/mcs.a004291

**Published:** 2019-12

**Authors:** Sonam Vyas, John N. Constantino, Dustin Baldridge

**Affiliations:** 1 St. Louis University School of Medicine, St. Louis, Missouri 63104, USA;; 2 Division of Child Psychiatry, Department of Psychiatry, Department of Pediatrics, Washington University School of Medicine, St. Louis, Missouri 63110, USA;; 3 Division of Genetics and Genomic Medicine, Department of Pediatrics, Washington University School of Medicine, St. Louis, Missouri 63110, USA

**Keywords:** aggressive behavior, attention deficit hyperactivity disorder, oppositional defiant disorder

## Abstract

Callous-unemotional (CU) traits are highly disabling behavioral characteristics, common predictors of delinquency and criminality, and pathognomonic for antisocial personality disorder. They are highly heritable, but their specific molecular genetic causes are unknown. Here, we briefly review the literature on neuropsychiatric correlates of 22q11.2 duplication and describe a newly identified case of a 737-kb microduplication within the low copy repeat (LCR) B-D region, involving a 13-yr-old early adoptee with mild developmental delay and severe, chronic antisocial behavior of early childhood onset. When psychiatric symptoms have been reported in relation to duplications in this specific region, 19% of the reports feature aggression—but never previously CU traits—as a component of the phenotype. We discuss the potential implications of gain of function in this chromosomal region for heritable origins of sociopathy and their possible relation to genetic influences on aggression.

## INTRODUCTION

Callous and unemotional (CU) trait profiles refer to consistent patterns of low emotional arousal, disregard for others, and lack of empathy. They are highly heritable and characterize individuals who go on to develop enduring patterns of antisocial behavior. Generally, conduct disorders of childhood encompass a range of antisocial behaviors, but the presence of CU traits identifies a subgroup of children who are particularly likely to manifest life-course-persistent antisocial personality and are more likely to engage in instrumental, predatory, or premediated aggression and harm to others (Viding et al. 2012). Individuals with substantial manifestations of these traits represent a substantial public health concern, as they require major resource allocations in health, social service, and education systems, exact serious harm in the community, are markedly overrepresented in the criminal justice system, and are generally minimally responsive to conventional therapies (Fontaine et al. 2011; Viding et al. 2012).

CU traits have been shown to be highly heritable (on the order of 0.80; Viding et al. 2008) and are presumed to be a function of polygenic liability (Dick et al. 2011; Rautiainen et al. 2016; Tielbeek et al. 2017). Candidate gene studies involving common allelic variation in the serotonin transporter gene *SLC6A4* (Sadeh et al. 2010; Cherepkova et al. 2016), the *COMT* gene (located in the 22q11 region outside of the duplication carried by this patient), and the *MAOA* gene have shown significant, if inconsistent, associations with antisocial outcome (Fowler et al. 2009; Hirata et al. 2013). Rare variants in MAOA have been associated with aggression (Brunner et al. 1993), but we are not aware of any prior report of rare variants associated with extreme CU traits.

The 22q11.2 duplication has been anecdotally linked to aggression, although the genotype–phenotype relationship has not been fully established because there are few detailed reports of aggression and a significant amount of phenotypic variability. Other reported features of this duplication include cardiac, genital, and facial abnormalities as well as a range of neuropsychiatric manifestations including autism, attention deficit/hyperactivity disorder (ADHD), developmental delays, obsessive–compulsive disorder (OCD), and schizophrenia (Olsen et al. 2018). The history of aggression and neuropsychiatric impairment associated with duplications in this chromosomal region and the remarkable severity of CU symptomatology in our patient motivated the report of this case, along with a detailed review of the association of duplications in this region with aggression. Here we present a patient who displayed profound CU traits from the time of early childhood and was found to have an atypical 737-kilobase (kb) 22q11.21 microduplication.

## RESULTS

### Clinical Presentation and Family History

The patient is currently a prepubertal 13-yr-old male whose behavioral abnormalities are so severe that he is in long-term residential care for intractable antisocial behavior. He was born full-term at 8 lbs 8 oz to a 32-yr-old mother. There is no genetic or phenotypic information available for either parent, because at age 4 mo, the patient was placed in foster care for substantiated neglect, and subsequently adopted at age 15 mo by parents who have cared for him and retained custody since. The foster home was documented to be supportive, and although the adoptive parents divorced during the child's early school years, there were no substantiated reports of abuse or neglect following his foster care placement at age 4 mo.

#### Psychiatric History

The propensity of aggression in this patient was severe and was noted by the adoptive family since the patient started living with them. His adoptive parents sought out clinical support by 2 yr of age and never resorted to physical discipline to modify his behaviors. He was placed in special education classrooms in a well-resourced school district from the time of early school age. At 6 yr of age the patient was known to display behaviors such as punching holes in his bedroom wall, tearing out the drywall, and destroying his clothes and bedsheets. He also hit and bit other children at school and was defiant toward teachers. At 7 yr of age, he began displaying cruelty to animals. Socially, he routinely violated the rights of others, struggled to empathize with peers, and displayed grossly inappropriate boundaries. On a standardized Inventory of Callous Unemotional Traits (ICU), he scored four standard deviations above the mean for an age-matched control population (Moore et al. 2017).

He was diagnosed with ADHD at age 3 yr. Other behavioral abnormalities included a tendency to gorge himself with food (since he was first adopted) and pica. His desire for food often triggered aggression. He never met diagnostic criteria for a major mood or psychotic disorder. The patient had two hospitalizations, at the ages of 9 and 11, because of aggression and behavioral problems.

The patient failed trials of numerous classes of psychotropic medication, including stimulants, α agonists, antidepressants, and all categories of mood stabilizers (neuroleptics, anticonvulsants, and lithium). Because he could not be safely managed at home or at school and despite extensive efforts to support him with both special education intervention and in-home support services, he was placed in residential care at age 11 yr, where he continued to exhibit intermittent antisocial behavior, including planned, predatory assault of female staff, as well as possible assault and threats toward multiple roommates.

#### Developmental History

In early childhood, the patient manifested an array of mild developmental delays. He walked at 18 mo, and at 30 mo a Cognitive Adaptive Test/Clinical Linguistic and Auditory Milestone Scale (CAT/CLAMS) demonstrated a developmental quotient (DQ) of 82. The patient did not use phrase speech until 4 yr. He displayed learning difficulties, and psychoeducational testing indicated that he was non-intellectually disabled.

#### Family History

Information about this patient's family history is limited as the patient was adopted. There is a strong family history of schizophrenia, including in the biological mother, the maternal aunt, and the maternal grandfather.

#### Physical and Mental Status Examination (Age 12 yr)

Physical examination was remarkable only for myopia and subtle phonological abnormalities. He has mild down slanting palpebral fissures, but no other appreciable craniofacial abnormalities. His neurological exam was entirely nonfocal. On mental status exam, the patient is moderately fidgety, aloof, and unable to verbalize insight about the consequences of his antisocial behaviors. His play is immature; he indicates that he would like to have “more friends,” but is unable to relate the absence of friendships to his behavior. His capacity for turn-taking conversation was limited to very concrete exchanges. He does not manifest any psychotic, obsessive, suicidal, or homicidal symptoms and does not seem particularly distressed about being in institutional care.

### Genomic Analysis

A chromosomal microarray analysis (CMA) using FirstStep^Dx^ PLUS by Lineagen commercial diagnostic laboratory was conducted using a buccal sample when the patient was 10 yr 10 mo old. FirstStep^Dx^ PLUS incorporates 2,784,985 probes and was designed for use in patients with neurodevelopmental disorders. Chromosome Analysis Suite software (manufactured by Affymetrix) was used to interpret data (Hensel et al. 2017). Copy-number changes (CNCs) that may not have been reported include duplications of <400 kb and deletions of <50 kb.

**Figure 1.**
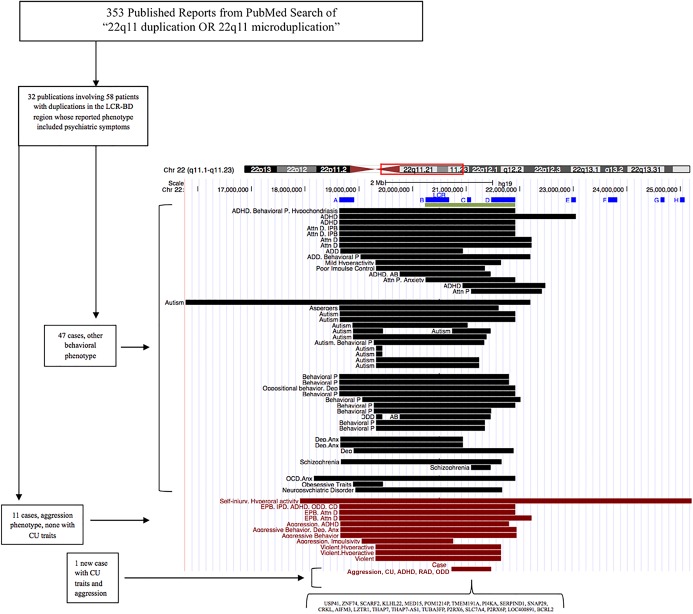
Visual summary of published literature of 22q11 duplication reports, including associated clinical features and overlap with LCR-BD region. Blue bars indicate LCRs. The green bar indicates LCR-BD. Black and red bars indicate lengths of duplications, and each bar is a unique case. These data were visualized using the custom annotation track feature of the UCSC Genome Browser (http://genome.ucsc.edu). (LCR) low copy repeat, (AB) abnormal behavior, (Anx) anxiety, (Attn D) attention deficit, (Attn P) attention problems, (Behavioral P) behavioral problems, (CD) conduct disorder, (CU) callous-unemotional traits, (Dep) depression, (EPB) external problem behavior, (IPB) internal problem behavior, (ODD) oppositional defiant disorder, (RAD) reactive attachment disorder.

A CNC of 22q11.21 gain (duplication) was detected (Table 1). The area of duplication is 737 kb, and there are 21 genes in the area (Fig. 1). The genomic coordinates are 20,728,956-21,465,662 (hg build 19). Using these determined coordinates, it appears that this duplication is flanked by LCRB and LCRD, without including either. No other CNCs of significance were detected by CMA, and fragile X testing was normal. Because this patient was adopted, information from biological family members was unavailable.

**Table 1.** Variant table

Variant type	Cytogenetic location	Size	Genomic coordinates	ClinVar accession ID
Copy-number gain (duplication)	22q11.21	737 kb	Chr 21:20,728,956-21,465,662 (hg19)	SCV000994926

This patient's duplication largely overlaps with the classical 3-Mb 22q11.21 duplication syndrome (OMIM #608363), although it is smaller and notably does *not* include the *TBX1* gene that has been implicated as a major cause of the phenotypic features of the 22q11.21 duplication syndrome.

## DISCUSSION

Here, we observed a 22q11.2 duplication in a child with an extremely unusual and sustained syndrome of antisocial behavior featuring pronounced CU traits from the time of early childhood. Our review indicated that clinical-level neuropsychiatric syndromes are described in approximately one-fourth of all 22q11.2 duplications involving the LCB-D region, and that 19% of these are associated with substantial aggression (Fig. 1).

Our patient was placed in foster care for neglect at the age of 4 mo, adopted at age 15 mo, and experienced the divorce of his parents during early school age (long after he first manifested severe antisocial behavior). From a genetic standpoint, and as summarized in Figure 1, he shared near-complete overlap with numerous previously reported duplication cases in which other neuropsychiatric syndromes have been described, including nearly all in which clinical-level aggression has been reported. It is possible that his remarkable presentation of CU traits arose from a combination of his specific chromosomal rearrangement, the course of his early experience, and background genetic factors unrelated to the 22q11.2 region. Although the role of this CNC cannot be assumed from a single case, the facts, however, that the patient manifested developmental and neuropsychiatric problems previously described for this duplication, that his duplication was atypical and involved only a small number of genes, and that his syndrome of antisocial behavior was so remarkable and uniquely characterized by severe CU traits prompt this report. There currently exist few clues to the molecular genetic structure of CU traits, which exact an overwhelming and nearly inestimable public health burden; genes in this region should be considered carefully as potential contributors to this devastating form of human social deviance.

## METHODS

A PubMed search using the terms “22q11 duplication OR 22q11 microduplication” yielded 353 total published reports that could be reviewed. After an extensive search through these papers, 32 publications, with the addition of one Decipher case, yielded 58 patients who had duplications that overlapped the proband's genetic duplication and documented clinical neuropsychiatric phenotypes (Supplemental Table 1). Eleven of these cases included aggression (10 of 11 completely overlapping our patient's duplication), but none specifically have documented CU traits.

## ADDITIONAL INFORMATION

### Data Deposition and Access

Copy-number variant details have been deposited in ClinVar (https://www.ncbi.nlm.nih.gov/clinvar/) under accession number SCV000994926.

### Ethics Statement

Informed consent for research and publication was provided by the patient's mother through written documentation. This case report was determined not to constitute Humans Subjects Research, and so further IRB approval was not required.

### Acknowledgments

We wish to acknowledge the patient and his family for their participation in the accrual of data for this report.

### Funding

Research reported in this manuscript was supported by the National Institutes of Health under award numbers U54 HD087011 (J.N.C., D.B.), K12 HL120002 (D.B.), and K08 HG010154 (D.B.). The content is solely the responsibility of the authors and does not necessarily represent the official views of the National Institutes of Health.

### Competing Interest Statement

The authors have declared no competing interest.

## Supplementary Material

Supplemental Material
