# Supplementary material for: 22q11.2 duplication: a review of neuropsychiatric correlates and a newly observed case of prototypic sociopathy
Source: Cold Spring Harb Mol Case Stud. 2019 Dec;5(6):a004291. doi: 10.1101/mcs.a004291 (PMC6913156; doi:10.1101/mcs.a004291)
Supplement: Supplemental Material [file supp_5_6_a004291__index.html]

Supplemental Material 

# 22q11.2 duplication: a review of neuropsychiatric correlates and a newly observed case of prototypic sociopathy

## Supplemental Material

- Supplemental\_Table\_1.xlsx
